# Quality of life outcomes after deep brain stimulation in acquired dystonia: a systematic review and meta-analysis

**DOI:** 10.1007/s10072-023-07106-y

**Published:** 2023-10-10

**Authors:** Adilijiang Aihemaitiniyazi, Huawei Zhang, Yue Hu, Tiemin Li, Changqing Liu

**Affiliations:** 1 Department of Neurosurgery, Sanbo Brain Hospital, Capital Medical University, Beijing, China; 2 Department of Neurosurgery, Aviation General Hospital, China Medical University, Beijing, 100012 China; 3 Department of Neurosurgery, Chongqing Sanbo Jiangling Hospital, Chongqing, China

**Keywords:** Quality of life, Deep brain stimulation, Dystonia

## Abstract

**Background:**

Dystonia is a condition that affects the ability to control the movement and function of the body’s muscles. It can cause not only physical problems, but also mental problems, resulting in impaired health-related quality of life (HRQoL). However, the effect of deep brain stimulation on quality of life in acquired dystonia remains unclear.

**Methods:**

We conducted a systematic literature review from January 2000 to October 2022，determined the eligible studies, and performed a meta-analysis of HRQoL outcomes based on the Short-Form Health Survey-36 (SF-36) after DBS to evaluate the effects of DBS on physical and mental QoL.

**Results:**

A total of 14 studies met the inclusion criteria and were systematically reviewed. A comprehensive meta-analysis was performed for 9 studies that reported physical and psychological data or physical component summary (PCS), or mental component summary (MCS) for SF-36. The mean (SD) age at DBS implantation was 34.29 (10.3) years, and the follow-up period after implantation was 2.21 (2.80) years. The random effects model meta-analysis revealed that both physical and mental domains of the SF-36 improved following DBS. There was no statistically significant difference between the physical domains (effect size=1.34; *p*<0.0001) and the mental domains (effect size=1.38; *p*<0.0001).

**Conclusion:**

This is the first meta-analysis that demonstrates significant benefits in HRQoL following DBS in patients with acquired dystonia. There were significant improvements in both physical QoL and mental QoL.

Dystonia is a neurological condition characterized by abnormal involuntary movements causing abnormal, often repetitive, movements, postures, or both [1]. In addition to abnormal movements and postures, movement disorders are often accompanied by nonmotor comorbidities, such as impairments in cognition, communication, nutritional intake, and sleep. This also increases the psychological burden of patients and seriously reduces their quality of life.

Previous classification methods for dystonia have focused more on etiology, and are divided into primary dystonia (with or without a hereditary pattern) and secondary dystonia (with other hereditary neurological conditions or due to known environmental cause), and psychological forms of dystonia [2]. With further understanding of the etiology and pathophysiological mechanisms of dystonia, the meaning of these terms has changed. According to the new classification method in 2013, in etiology axis dystonia is subdivided into inherited, acquired, and idiopathic dystonia [3]. Dystonia is defined as being acquired when it is non inherited and has a known acquired or exogenous origin. The causes of acquired dystonia are varied, including infections, traumatic brain injury, drugs or drugs, etc. [3].

Management of dystonia is particularly challenging, because pharmacological treatment is often unsatisfactory, or side effects are dose-limiting factors [4]. In patients with medically refractory dystonia, deep brain stimulation (DBS) of the globus pallidus internus (GPi) has been shown to be an effective and safe treatment [5–8]. However, the main outcome index of most studies was the improvement of motor function, and few studies recorded nonmotor symptoms in dystonia. A recent literature review reported that DBS can relieve the dystonic pain [9], and has a major impact on mood, anxiety, and cognition [10]. This indicate that DBS may also be beneficial in HRQoL following DBS in patients with dystonia. A previous systematic review study has discussed the effect of DBS on dystonia on the improvement of HRQoL [11]. However, due to the small number of relevant studies and limited quality, no further meta-analyses were performed on quality of life improvement across dystonia subtypes. In recent years, a number of studies have explored the effect of DBS on patients with acquired dystonia [12–15]. There is a need for an update and quantitative analyses on a systematic review of HRQoL outcomes after DBS in acquired dystonia.

In view of the small numbers of patients, the substantial variability in improvement of quality of life, and the considerable clinical heterogeneity of patients with acquired dystonia [12–15], we investigated the effects of DBS on patients with acquired dystonia, in a meta-analysis of published patient data. The aim was to assess the improvement of the quality life outcomes average response to DBS in these patients and to isolate outcome predictors in a larger cohort.

## Methods

This systematic review was conducted according to the Preferred Reporting Items for Systematic Reviews and Meta-Analyses (PRISMA) checklist [16]. A protocol was prospectively registered with PROSPERO (CRD42022362490).

### Eligibility criteria

In this systematic review and meta-analysis, we included articles fulfilling the following inclusion criteria: (1) a cohort of patients with acquired dystonia; (2) DBS targeted to the GPi, STN, or thalamus; (3) reporting both motor and HR-QoL outcomes using formal assessment scales; and (4) papers published in English. Review articles, meta-analyses and conference papers were excluded. In addition, we explored additional articles by reviewing the reference lists of included articles.

### Literature search

According to the PRISMA guidelines a literature search of articles published from January 2000 to October 2022 was performed using PubMed, Web of Science and the Cochrane Library, restricted to the English language. The search terms included dystonia, secondary dystonia, acquired dystonia, tardive dystonia, dyskinetic cerebral palsy, deep brain stimulation, DBS, pallidal stimulation, quality of life, QoL and HR-QoL. Two investigators (AA and HW) independently screened for duplicates, screened the titles and abstracts, and then reviewed full texts based on the inclusion criteria. Disagreements were resolved through discussion.

### Data extraction

We collected the following information for each study: a type of study, number of patients, gender, DBS targets, types of dystonia before DBS, age at DBS, disease duration before DBS, follow-up period after DBS, preoperative and postoperative scores of dystonia rating scales and HR-QoL outcomes.

### Statistical analysis

We analyzed preoperative and postoperative HRQOL means and SD, sample sizes and preoperative and postoperative correlation values through Comprehensive Meta-Analysis. To determine heterogeneity, we conducted meta-analysis variability tests: Cochran’s Q, and I^2^ statistics [17, 18]. A random effects model meta-analysis technique was applied to provide weighted individual effect sizes as well as an overall effect size based on the standardized mean difference of the comparisons [19, 20]. Furthermore, publication bias was assessed by funnel plot asymmetry and Egger’s regression test [21].

## Results

### Literature search

**Fig. 1:**
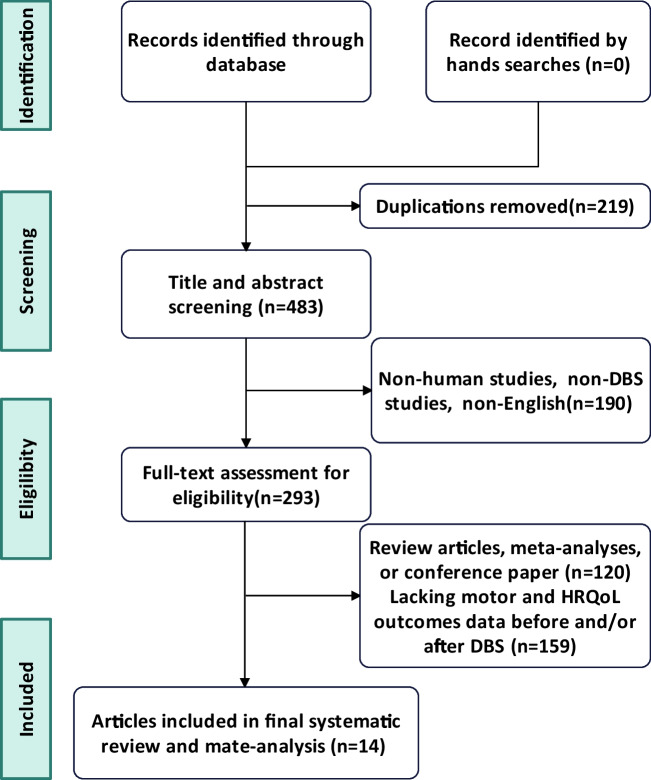
PRISMA flow diagram of outlining the search strategy and results based on screening the titles and full text of articles

This systematic review and meta-analysis search identified a total of 702 records (Fig. 1). After removing duplicated articles, we performed title and abstract screening and then examined the full texts of 293 remaining articles. Screening was conducted according to the inclusion criteria, and identified 14 articles that reported preoperative and postoperative motor and HR-QoL outcome data following DBS in patients with Acquired dystonia(Table 1).

**Table 1:** Quality of life outcomes after DBS in patients with acquired (secondary) dystonia

No	Author	year	study design	participants(n)	Gender (male/ female)	Age at surgery (years)	Disease duration before surgery (years)	Target	Follow-up period(yeasr)	Motor improvement at the last follow-up	QOL items with significant improvement after DBS
1	Pretto et al. [19]	2008	Prospective single centre study	9	6/3	44.4 ± 24.3	NA	Bil GPI	0.5	BFMDRS-M 44.6%	EQ-5D
2	Vidailhet et al. [20]	2009	prospective multicentre pilot study	13	4/9	30.2 ± 8.6	30.2 ± 8.6	Bil GPI	1	BFMDRS-M 24.4%	SF-36
3	Gruber et al. [21]	2009	Retrospective single centre study	9	2/7	63.2 ± 12.5	5.3 ± 3.1	Bil GPI	3.39 ± 1.67	BFMDRS-M 83.3%	SF-36
4	Kim et al. [22]	2011	Retrospective single centre study	15	10/5	29.1 ± 5.7	23.3 ± 9.0	Bil GPI	2.89 ± 1.85	BFMDRS-M 31.5%	SF-36
5	Gimeno et al. [23]	2011	Retrospective single centre study	6	5/1	9.8 ± 3.1	9.8 ± 3.1	Bil GPI	0.5，1	No significant changes	CPCHILD
6	Romitoa et al. [24]	2014	Retrospective single centre study	15	7/8	29.8 ± 9.5	25.4 ± 8.6	Bil GPI	4.4 ± 1.8	BFMDRS-M 50%; BFMDRS-D 30%	SF-36
7	Pouclet-Courtemanche et al. [25]	2016	Prospective single centre study	19	5/14	51.8 ± 12.6	6.3 ± 8.1	Bil GPI	0.5	AIMS 60%	Lehman Quality of Life Interview
8	Deng et al. [26]	2017	Retrospective single centre study	10	6/4	29.8 ± 15.4	5.5 ± 2.5	Bil STN	5.46 ± 2.53	BFMDRS-M 55.9 ± 28.3% and BFMDRS-D 62.6 ± 32.0%	SF-36
9	Doreen et al. [27]	2018	prospective randomised multicentre study	12	2/10	62.0 ± 11.1	6.2 ± 5.6	Bil GPI	0.25，0.5	BFMDRS-M 22.8%	SF—36
10	Kim JH et al. [28]	2019	Retrospective single centre study	12	6/6	29.3 ± 5.7	29.3 ± 5.7	Bil GPI	1	BFMDRS-M 37.27%	SF—36
11	San Luciano et al. [29]	2021	prospective single centre study	4	2/2	14.5 ± 3.7	13 ± 4.7	Bil Vop/Vim DBS	0.5，1	BFMDRS-M 21.5%	PedsQL
12	Mandarano et al. [30]	2022	Retrospective single centre study	9	7/2	17.42 ± 13	17.42 ± 13	Bil GPI	1	BFMDRS-M 19.9%	CHQ-PF50
13	Krause et al. [31]	2022	Retrospective single centre study	7	1/6	57.6 ± 17.4	5.43 ± 4.03	Bil GPI	10.2 ± 2.8	BFMDRS-M 73%	SF—36
14	Koy et al. [32]	2022	Prospective randomised Multicenter Study	16	10/6	13.4 ± 2.9	13.4 ± 2.9	Bil GPI	0.25，0.5，0.75.1	No significant changes	CPCHILD，SF-36

To assess the HRQoL outcomes, Short Form Health Survey-36 (SF-36) [33] was the most commonly used instrument (9 articles). The Child Health Index of Life with Disabilities questionnaire (CPCHILD) was used in 2 studies [34], the Euro-QoL (EQ-5D) in one study [35], Lehman Quality of Life Interview in 1 study [36], and the Pediatric Quality of Life Inventory (PedsQL) in 1 study [37]. Quality of life scores other than SF-36 were not included in the final meta-analysis because these were poorly reported, with insufficient details on changes in scores.

### Demographics of the cohort

Of the fourteen studies included, two were prospective multicenter randomized clinical trials [15, 30]; three were prospective single-center studies [12, 22, 28]; one was a prospective multicenter study [23]; and eight were retrospective single-center studies [13, 14, 24–27, 29, 31]. In the prospective randomized multicenter study, quality of life outcomes were available only for patients allocated to the deep brain stimulation group, both before and after DBS treatment.

Thus, the final cohort comprised 156 patients with 73 males and 83 females; mean (SD) age at DBS implantation, 34.29 (10.3) years; disease duration before implantation, 15.58 (6.07) years; and follow-up period after implantation, 2.21 (2.80) years.

### Results of the meta-analysis

This comprehensive meta-analysis investigated HR-QoL outcomes after DBS implantation in patients with acquired dystonia. Synthesized data from nine longitudinal studies included preoperative and postoperative longitudinal data of the eight domains of SF-36 as well as PCS and MCS, which totaled 64 comparisons. We conducted a random effects model meta-analysis on the 64 comparisons and found a significant overall standardized mean difference statistic equal to 1.36, SE=0.13, 95%CI=1.10–1.63, *p*<0.05, Z=10.12. According to these results, quality of life scores were higher after DBS implantation at baseline.

### Heterogeneity and publication bias

**Fig. 2:**
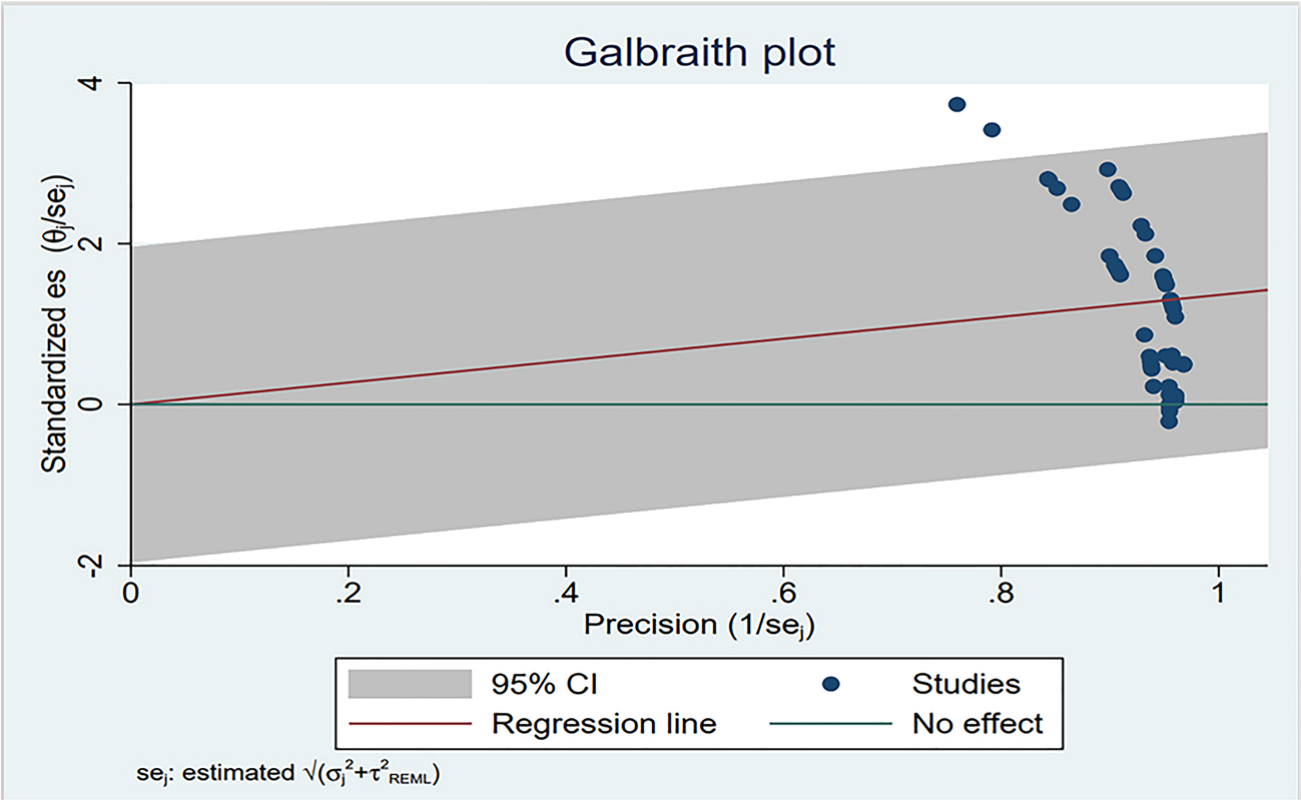
Galbraith plots (assessing heterogeneity)

**Fig. 3:**
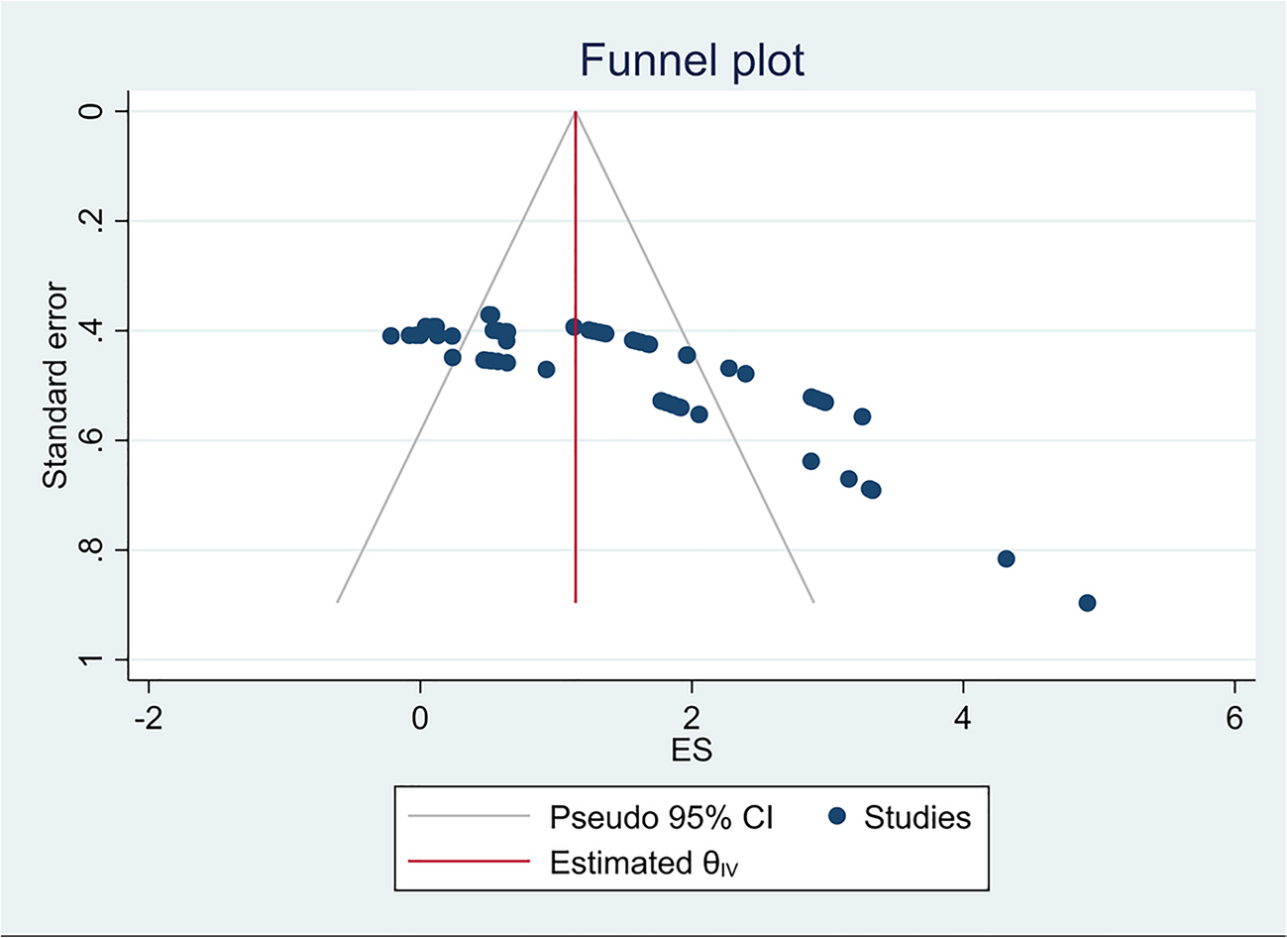
Funnel plot of SE by standard difference in means

The three meta-analysis variability tests indicated mild heterogeneity in the 64 comparisons: (1) Cochran’s *Q*=300.41 *p*=0.000; (2) Tau squared (T2)=0.930; and (3) Higgins and Green’s I2 = 81.89%.(4)Galbraith plot (Fig. 2). These values point to homogenous HRQoL and DBS comparisons. Consistent with classic meta-analysis techniques for evaluating publication bias, we created and evaluated a funnel plot (Fig. 3). Inspection indicates that symmetry is evident. The publication bias test evaluated 64 comparisons by determining the relationship between effect size and accuracy. The regression result of Egger’s test shows that there is a significant intercept (*p*≤0.0001). Thus, random effects model meta-analyses were applied. We are therefore cautious to conclude that overall publication bias is within acceptable guidance.

### Physical and mental domains: moderator variable analysis

**Fig. 4:**
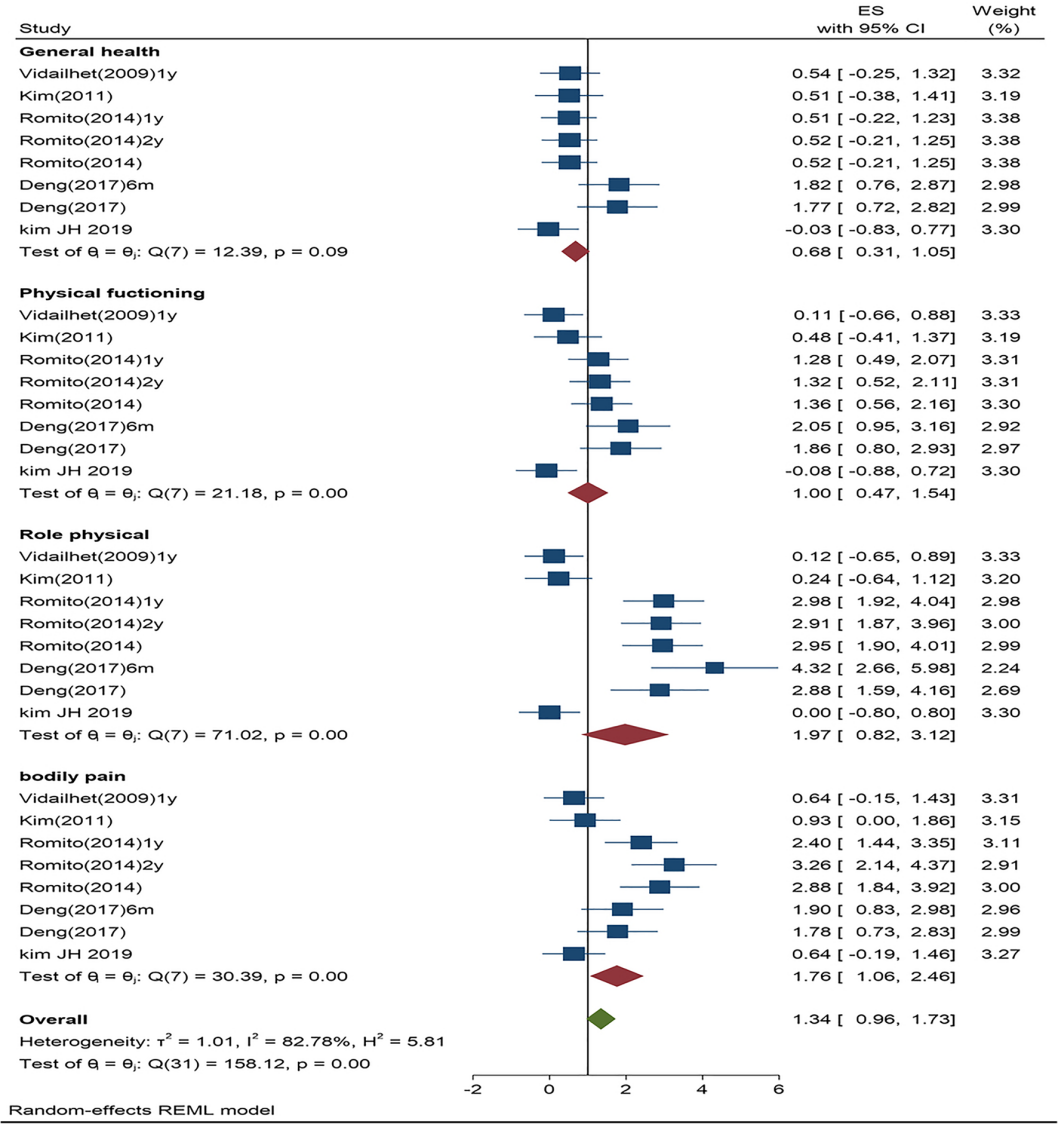
Physical domain forest plot showing summary data as individual weighted effect sizes (standardized mean difference) and 95% CIs

**Fig. 5:**
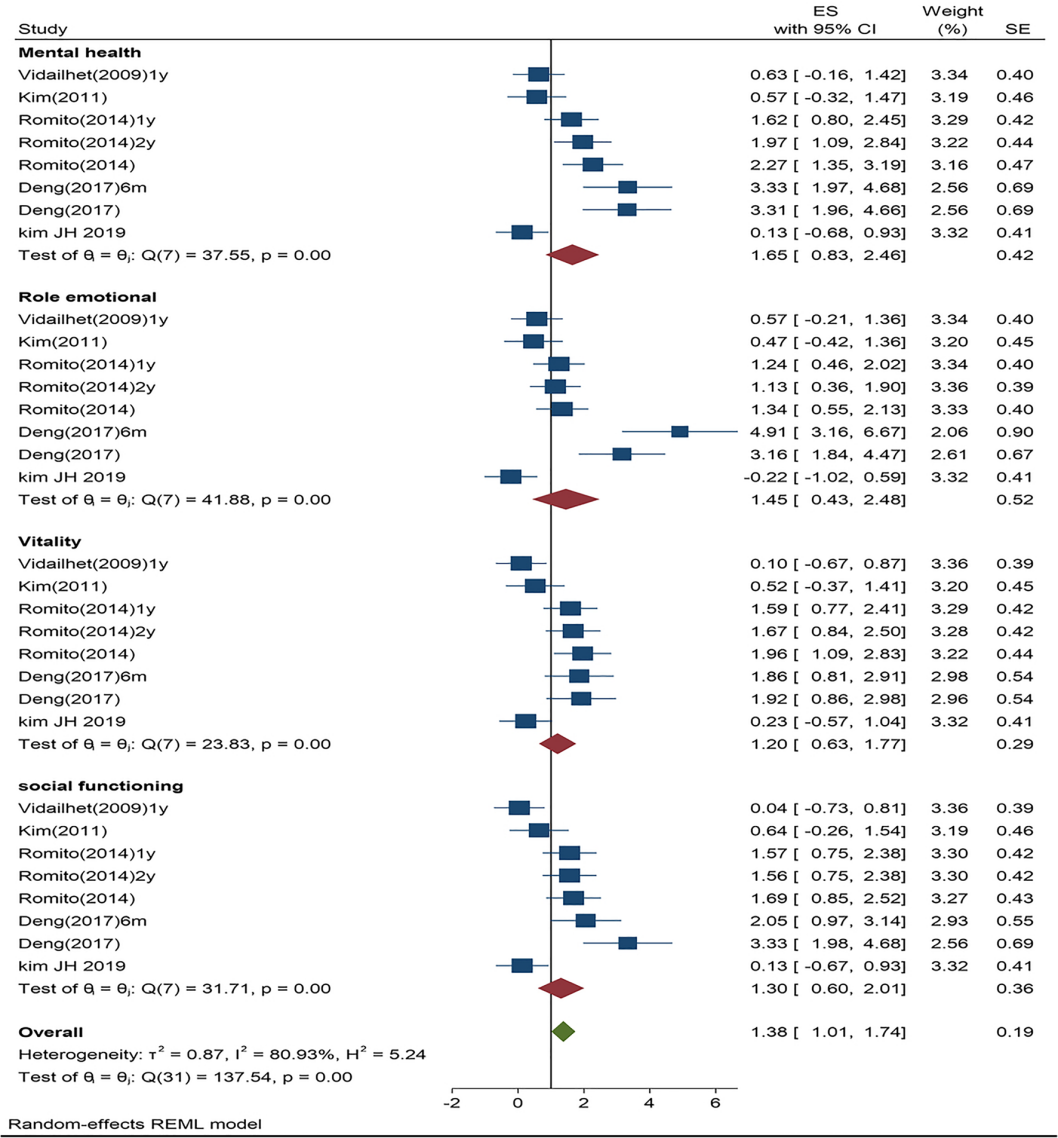
Mental domain forest plot showing summary data as individual weighted effect sizes (standardized mean difference) and 95% Cis

Most importantly, to answer our primary question on the physical and mental domains of the HR-QoL, we further analyzed the individual scores for both subcategories. Consistent with the traditional approaches for analyzing the standardized mean effects for different subgroups, we identified a clear pattern of distinction. The 48 comparisons for each of the subcategories indicated different effect sizes: (1) physical effect size=1.35, SE=0.20, 95%CI=0.96–1.74, *p*<0.0001, *Z*=6.83, *T*^2^ =1.02, *I*^2^ =83.20%; and (2) mental effect size=1.38, SE=0.19, 95%CI=1.01–1.74, *p*<0.0001, *Z*=7.41, *T*^2^ =0.87, *I*^2^=80.93%. To examine the individual studies standardized mean effects and the 95% CIs for each of the subcategories, we created two separate forest plots (Figs. 4 and 5). Noting that both of the effects for the physical and mental subcategories reached significance, we conducted a Z test using the means and variances of the estimated effects leading to a p value on the differences in the estimated effects between the two subcategories. For hypothesis testing, the true effect size is predicted to be the same for both subcategories, H0 : Effect size A (physical)=Effect Size B (mental). Rejecting the null hypothesis at the traditional alpha level (*p*<0.05) indicates different effect sizes for the two subcategories. A Z test, as outlined above, showed that the effect sizes between the physical and mental subcategories were significantly different (*p*=0.0015). This indicates that the physical subcategories rated higher than the mental subcategories. Thus, these patients with dystonia improved more on the physical aspects of the QoL than on the mental aspects.

Given the robust physical and mental subcategory findings, moderator variable analyses on the four specific questions for each domain revealed significant effect sizes for the subgroups within each domain. For the physical domains, the four significant standardized mean difference effects follow: (1) physical functioning=1.01; (2) role physical=1.98; (3) bodily pain=1.76; and (4) general health=0.69. Thus, each domain improved following DBS, and the role physical domain showed the largest effect size. Moderator variable analysis of the mental domains indicated four significant standardized difference mean effects: (1) vitality=1.20; (2) social functioning=1.30; (3) role emotional=1.45; and (4) mental health=1.65. Although the effect sizes were slightly smaller than those in the four physical domains, each of the subgroups in the mental domain showed medium to large magnitudes with vitality being largest.

## Discussion

DBS is a well-recognized and widely used intervention for the treatment of many of the most common forms of movement disorders, including PD, essential tremor and primary dystonia. It has been studied in acquired dystonia cases with variable results. We conducted review and meta-analysis of acquired dystonia cases treated with DBS described in the literature so far. Overall, the data referenced and discussed in this overview show a potential effectiveness of DBS for these acquired dystonia. Across the quality of life assessment measures, we found significant improvements in most quality of life scores, particularly in the SF-36 quality of life score, which is used most frequently in various studies, before and after DBS surgery. We found significant improvements in both the physical and mental areas. The areas of physical quality of life (physical function, physical role, physical pain, and general health) showed improvement, with large effect sizes ranging from 0.69 to 1.98. As the physical functioning and role physical domains assessed physical disability related to the disease, GPi DBS effectively improved these domains in close association with the reduction of dystonic symptoms. Our findings are consistent with previous findings, that is, the BFMDRS scale and the DIS scale were used to assess the degree of dystonia, which showed significant improvement in physical disability scores after DBS [5–8, 38]. Similarly, improvement of pain following DBS was reported based on pain-specific assessment questionnaires [10, 11, 39, 40]. Furthermore, a recent pooled meta-analysis showed that DBS improved pain based on the TWSTRS pain sub-scores in patients with cervical dystonia. In a recent meta-analysis [9], the results showed improvement in the pain rating scale across the cohort after DBS. Pain severity, pain frequency, and analgesic need were all reduced.

The mental QoL domains (vitality, social functioning, role emotional and mental health) showed improvement with medium to large effect sizes ranging from 1.20 to 1.65. Earlier studies reported various changes in depression and anxiety disorders with stable or improved symptoms after DBS surgery [41, 42]. Patients with acquired dystonia because long-term dystonia can lead to social disorders or mental disorders such as anxiety or depression. This can lead to poor follow-up treatment and even suicidal ideation. We should pay more attention to mental problems and screen for possible suicidal ideation in a timely manner. A multidisciplinary DBS team consisting of neurologists, neurosurgeons, psychiatrists, psychologists, rehabilitation therapists, etc., may help to conduct adequate preoperative assessment and obtain maximum benefit postoperatively [8, 43].

The included cases mainly included movement disorder cerebral palsy and delayed dystonia, or trauma-induced dystonia. Our analysis found no clear relationship between etiology and improvement in quality of life after surgery. This may be due to a long history of both cerebral palsy and tardive dystonia, and there may be severe structural abnormalities in the brain.

In the selection of DBS surgical targets, 12 research double-sided GPi nuclei, one STN nucleus and one Vop/Vim nucleus. No further statistical analyses were performed due to study and case number limitations. Many studies have confirmed that BP-GPi DBS can improve BFMDRS score and quality of life score, and is a relatively safe and effective target [5–8]. In a retrospective cohort study in China, bilateral STN-DBS stimulation was perform. The mean follow-up was 65.6 ± 30.4 months. BFMDRS exercise and disability scores improved further at the last follow-up, 87.3± 17.0% and 84.3% ± 22.9%, respectively, while AIMS scores improved by 88.4 ± 16.1%. Patients showed a statistically significant (*p* < 0.01) improvement in both physical and mental QoL, and this benefit persisted and stabilized [26]. In a phase 1 clinical trial, four cases of bilateral Vop/Vim DBS were reported. The results showed an average improvement of 21.5% in BFMDRS motor scores, 15.7% in BFMDRS disability subscale scores, and 27% in total PedsQL scores [29]. STN and Vop/Vim are very promising DBS targets. Further large-sample, randomized controlled studies are needed to compare the clinical effects of DBS targets in patients with acquired dystonia.

However, this review and meta-analysis has several limitations. First, research on the treatment of acquired dystonia with DBS is limited, with some studies not providing specific values for quality of life scores, and some articles only providing total PCS/MCS scores and not data for the eight domains of the SF-36. Second, only studies reporting SF-36 quality of life scores were included in the meta-analysis, and the rest of the quality of life scores were not included. Finally, most of the included studies were observational and unblinded. The inherent biases arising from patient selection and unblinded assessment could not be addressed.

## Conclusion

This systematic review and meta-analysis supports the premise that DBS significantly improves both physical and mental QoL along with motor improvement in patients with acquired dystonia. However, long-term data, STN DBS and Vop/Vim DBS data in this population remain scarce. Importantly, a unified reporting format including the mean, precision and range of the data is desirable for further accumulation of useful data. When evaluating the postoperative clinical effect of DBS, we should not only pay attention to the improvement of postoperative dyskinesia, but also observe the improvement of patients’ quality of life scores, which provides us with new clinical evaluation strategies. In addition, sensitive and disease-specific measures for different subtypes of dystonia need to be developed. Overall, the results of future studies will allow clinicians to provide a clearer view of patients and improve patients’ HRQoL after DBS.

## References

[1] Balint B, Mencacci NE, Valente EM, Pisani A, Rothwell J, Jankovic J, Vidailhet M, Bhatia KP (2018). Dystonia. Nat Rev Dis Primers.

[2] Fahn S, Eldridge R (1976). Definition of dystonia and classification of the dystonic states. Adv Neurol.

[3] Albanese A, Bhatia K, Bressman SB, Delong MR, Fahn S, Fung VS, Hallett M, Jankovic J, Jinnah HA, Klein C, Lang AE, Mink JW, Teller JK (2013). Phenomenology and classification of dystonia: a consensus update. Mov Disord.

[4] Jankovic J (2006). Treatment of dystonia. Lancet Neurol..

[5] Vidailhet M, Vercueil L, Houeto J-L (2007). Bilateral, pallidal, deep-brain stimulation in primary generalised dystonia: a prospective 3 year follow-up study. Lancet Neurol.

[6] Kupsch A, Benecke R, Müller J, Trottenberg T, Schneider GH, Poewe W, Eisner W, Wolters A, Müller JU, Deuschl G, Pinsker MO, Skogseid IM, Roeste GK, Vollmer-Haase J, Brentrup A, Krause M, Tronnier V, Schnitzler A, Voges J, Nikkhah G, Vesper J, Naumann M, Volkmann J (2006). Pallidal deep-brain stimulation in primary generalized or segmental dystonia. N Engl J Med.

[7] Volkmann J, Wolters A, Kupsch A (2012). Pallidal deep brain stimulation in patients with primary generalised or segmental dystonia: 5-year follow-up of a randomised trial. Lancet Neurol.

[8] Moro E, LeReun C, Krauss JK (2017). Efficacy of pallidal stimulation in isolated dystonia: a systematic review and meta-analysis. Eur J Neurol.

[9] Perides S, Lin JP, Lee G, Gimeno H, Lumsden DE, Ashkan K, Selway R, Kaminska M (2020). Deep brain stimulation reduces pain in children with dystonia, including in dyskinetic cerebral palsy. Dev Med Child Neurol.

[10] Eggink H, Szlufik S, Coenen MA, van Egmond ME, Moro E, Tijssen MAJ (2018). Non-motor effects of deep brain stimulation in dystonia: A systematic review. ParkinsonismRelatDisord.

[11] Tsuboi T, Wong JK, Okun MS, Ramirez-Zamora A (2020). Quality of life outcomes after deep brain stimulation in dystonia: A systematic review. Parkinsonism Relat Disord.

[12] San Luciano M, Robichaux-Viehoever A, Dodenhoff KA, Gittings ML, Viser AC, Racine CA, Bledsoe IO, Watson Pereira C, Wang SS, Starr PA, Ostrem JL (2020) Thalamic deep brain stimulation for acquired dystonia in children and young adults: a phase 1 clinical trial. J Neurosurg Pediatr, 10.3171/2020.7.PEDS20348 27(2):203–212 10.3171/2020.7.PEDS20348 PMC8155109 33254134

[13] Mandarano R, Danieli A, Petacchi E, Di Pede C, Mondani M, Armellin MT, Facchin D, Martinuzzi A (2022). Deep Brain Stimulation in childhood-onset dystonia due to brain pathology. A long-term study. Eur J Paediatr Neurol.

[14] Koy A, Kühn AA, Huebl J, Schneider GH, van Riesen AK, Eckenweiler M, Rensing-Zimmermann C, Coenen VA, Krauss JK, Saryyeva A, Hartmann H, Haeussler M, Volkmann J, Matthies C, Horn A, Schnitzler A, Vesper J, Gharabaghi A, Weiss D, Bevot A, Marks W, Pomykal A, Monbaliu E, Borck G, Mueller J, Prinz-Langenohl R, Dembek T, Visser-Vandewalle V, Wirths J, Schiller P, Hellmich M, Timmermann L (2022). STIM-CP investigators. Quality of Life After Deep Brain Stimulation of Pediatric Patients with Dyskinetic Cerebral Palsy: A Prospective, Single-Arm, Multicenter Study with a Subsequent Randomized Double-Blind Crossover (STIM-CP). Mov Disord.

[15] Krause P, Kroneberg D, Gruber D, Koch K, Schneider GH, Kühn AA (2022). Long-term effects of pallidal deep brain stimulation in tardive dystonia: a follow-up of 5-14 years. J Neurol.

[16] Moher D, Liberati A, Tetzlaff J (2010). Altman DG; PRISMA Group. Preferred reporting items for systematic reviews and meta-analyses: the PRISMA statement. Int J Surg.

[17] Sutton AJ, Higgins JPT (2008). Recent developments in meta-analysis. Stat Med.

[18] Duval S, Tweedie R (2000). Trim and fill: a simple funnel-plot-based method of testing and adjusting for publication bias in meta-analysis. Biometrics.

[19] Higgins JPT, Thompson SG, Spiegelhalter DJ (2009). A re-evaluation of random-effects meta-analysis. J R Stat Soc Ser A Stat Soc.

[20] Borenstein M, Higgins JPT (2013). Meta-Analysis and subgroups. Prev Sci.

[21] Sutton AJ, Duval SJ, Tweedie RL (2000). Empirical assessment of effect of publication bias on meta-analyses. BMJ.

[22] Pretto TE, Dalvi A, Kang UJ, Penn RD (2008). A prospective blinded evaluation of deep brain stimulation for the treatment of secondary dystonia and primary torticollis syndromes. J Neurosurg.

[23] Vidailhet M, Yelnik J, Lagrange C, Fraix V, Grabli D, Thobois S, Burbaud P, Welter ML, Xie-Brustolin J, Braga MC, Ardouin C, Czernecki V, Klinger H, Chabardes S, Seigneuret E, Mertens P, Cuny E, Navarro S, Cornu P, Benabid AL, Le Bas JF, Dormont D, Hermier M, Dujardin K, Blond S, Krystkowiak P, Destée A, Bardinet E, Agid Y, Krack P, Broussolle E, Pollak P; French SPIDY-2 Study Group. Bilateral pallidal deep brain stimulation for the treatment of patients with dystonia-choreoathetosis cerebral palsy: a prospective pilot study. Lancet Neurol. 2009 Aug;8(8):709-17. doi: 10.1016/S1474-4422(09)70151-6. Epub 2009 Jul 1. 10.1016/S1474-4422(09)70151-6 19576854

[24] Gruber D, Trottenberg T, Kivi A, Schoenecker T, Kopp UA, Hoffmann KT, Schneider GH, Kühn AA, Kupsch A (2009). Long-term effects of pallidal deep brain stimulation in tardive dystonia. Neurology.

[25] Kim JP, Chang WS, Chang JW (2011). Treatment of secondary dystonia with a combined stereotactic procedure: long-term surgical outcomes. Acta Neurochir (Wien).

[26] Gimeno H, Tustin K, Selway R, Lin JP (2012). Beyond the Burke-Fahn-Marsden Dystonia Rating Scale: deep brain stimulation in childhood secondary dystonia. Eur J Paediatr Neurol.

[27] Romito LM, Zorzi G, Marras CE, Franzini A, Nardocci N, Albanese A (2015). Pallidal stimulation for acquired dystonia due to cerebral palsy: beyond 5 years. Eur J Neurol.

[28] Pouclet-Courtemanche H, Rouaud T, Thobois S, Nguyen JM, Brefel-Courbon C, Chereau I, Cuny E, Derost P, Eusebio A, Guehl D, Laurencin C, Mertens P, Ory-Magne F, Raoul S, Regis J, Ulla M, Witjas T, Burbaud P, Rascol O, Damier P (2016). Long-term efficacy and tolerability of bilateral pallidal stimulation to treat tardive dyskinesia. Neurology..

[29] Deng ZD, Li DY, Zhang CC, Pan YX, Zhang J, Jin H, Zeljec K, Zhan SK, Sun BM (2017). Long-term follow-up of bilateral subthalamic deep brain stimulation for refractory tardive dystonia. Parkinsonism Relat Disord.

[30] Gruber D, Südmeyer M, Deuschl G, Falk D, Krauss JK, Mueller J, Müller JU, Poewe W, Schneider GH, Schrader C, Vesper J, Volkmann J, Winter C, Kupsch A (2018). Schnitzler A; DBS study group for dystonia. Neurostimulation in tardive dystonia/dyskinesia: A delayed start, sham stimulation-controlled randomized trial. Brain Stimul.

[31] Kim JH, Jung NY, Chang WS, Jung HH, Cho SR, Chang JW (2019). Intrathecal Baclofen Pump Versus Globus Pallidus Interna Deep Brain Stimulation in Adult Patients with Severe Cerebral Palsy. World Neurosurg.

[32] Brazier JE, Harper R, Jones NM, O'Cathain A, Thomas KJ, Usherwood T, Westlake L (1992). Validating the SF-36 health survey questionnaire: new outcome measure for primary care. BMJ.

[33] Narayanan UG, Fehlings D, Weir S, Knights S, Kiran S, Campbell K (2006). Initial development and validation of the Caregiver Priorities and Child Health Index of Life with Disabilities (CPCHILD). Dev Med Child Neurol..

[34] Hurst NP, Kind P, Ruta D, Hunter M, Stubbings A (1997). Measuring health-related quality of life in rheumatoid arthritis: validity, responsiveness and reliability of EuroQol (EQ-5D). Br J Rheumatol..

[35] Lehman A (1988). A quality of life Interview for the chronically mentally ill. Eval. Program Plann.

[36] Varni JW, Seid M, Kurtin PS (2001). PedsQL 4.0: reliability and validity of the Pediatric Quality of Life Inventory version 4.0 generic core scales in healthy and patient populations. Med Care.

[37] Kiss ZH, Doig-Beyaert K, Eliasziw M, Tsui J, Haffenden A (2007). Suchowersky O; Functional and Stereotactic Section of the Canadian Neurosurgical Society; Canadian Movement Disorders Group. The Canadian multicentre study of deep brain stimulation for cervical dystonia. Brain.

[38] Volkmann J, Mueller J, Deuschl G, Kühn AA, Krauss JK, Poewe W, Timmermann L, Falk D, Kupsch A, Kivi A, Schneider GH, Schnitzler A, Südmeyer M, Voges J, Wolters A, Wittstock M, Müller JU, Hering S, Eisner W, Vesper J, Prokop T, Pinsker M, Schrader C, Kloss M, Kiening K, Boetzel K, Mehrkens J, Skogseid IM, Ramm-Pettersen J, Kemmler G, Bhatia KP, Vitek JL, Benecke R (2014). DBS study group for dystonia. Pallidal neurostimulation in patients with medication-refractory cervical dystonia: a randomised, sham-controlled trial. Lancet Neurol.

[39] Walsh RA, Sidiropoulos C, Lozano AM, Hodaie M, Poon YY, Fallis M, Moro E (2013). Bilateral pallidal stimulation in cervical dystonia: blinded evidence of benefit beyond 5 years. Brain.

[40] Skogseid IM, Ramm-Pettersen J, Volkmann J, Kerty E, Dietrichs E, Røste GK (2012). Good long-term efficacy of pallidal stimulation in cervical dystonia: a prospective, observer-blinded study. Eur J Neurol..

[41] Jahanshahi M, Torkamani M, Beigi M, Wilkinson L, Page D, Madeley L, Bhatia K, Hariz M, Zrinzo L, Limousin P, Ruge D, Tisch S (2014). Pallidal stimulation for primary generalised dystonia: effect on cognition, mood and quality of life. J Neurol.

[42] Lozano AM, Lipsman N, Bergman H, Brown P, Chabardes S, Chang JW, Matthews K, McIntyre CC, Schlaepfer TE, Schulder M, Temel Y, Volkmann J, Krauss JK (2019). Deep brain stimulation: current challenges and future directions. Nat Rev Neurol.

[43] Giugni JC, Okun MS (2014). Treatment of advanced Parkinson's disease. Curr Opin Neurol.

